# Mechanisms of fluoroquinolone resistance among *Escherichia coli* isolates from urinary tract infections in Thailand

**DOI:** 10.1371/journal.pone.0325175

**Published:** 2025-05-30

**Authors:** Nipaporn Tewawong, Siriporn Kowaboot, Warangkana Lektrakul, Utsanee Supcharoengoon, Naiyana Watanagul, Pannamthip Pitaksajjakul

**Affiliations:** 1 Faculty of Medical Technology, Rangsit University, Pathum Thani, Thailand; 2 Department of Microbiology, Nopparat Rajathanee Hospital, Khannayao, Bangkok, Thailand; 3 Department of Social and Environmental Medicine, Faculty of Tropical Medicine, Mahidol University, Bangkok, Thailand; 4 Center of Excellence for Antibody Research, Faculty of Tropical Medicine, Mahidol University, Bangkok, Thailand; Universidade Catolica Portuguesa, PORTUGAL

## Abstract

*Escherichia coli* is the major causative agent for urinary tract infections (UTIs), and fluoroquinolones (FQ) are commonly used in the treatment of patients with UTIs. The surge in FQ-resistant *E. coli* is an important public health threat worldwide. We investigated the prevalence and mechanisms of FQ resistance among FQ-resistant *E. coli* isolated from UTI patients. A total of 131 FQ-resistant *E. coli* strains were characterized and broth microdilution assay showed that 108 strains (82.4%) were highly resistant to ciprofloxacin (MIC ≥ 32 μg/mL). All strains were analyzed for plasmid-mediated quinolone resistance (PMQR) genes, with 37 (28.2%) testing positive. Among the PMQR genes detected, *aac(6’)-Ib-cr* was the most frequent, found in 30 strains (22.9%), followed by *qnrS* in 10 strains (7.6%) and *qnrB* in 1 strain (0.8%). PCR assay showed that all carried *acrA*, *acrB,* and *tolC* genes, but 33 strains (25.2%) revealed at least 4-fold reduction in ciprofloxacin MIC when using PAβN and CCCP as efflux pump inhibitors, indicating the role of the AcrAB efflux pump in ciprofloxacin resistance in these strains. The 19 strains of high-level ciprofloxacin-resistant *E. coli* were selected to determine the target enzyme alteration by PCR assay and DNA sequencing. Genetic analysis revealed that 16 strains (84.2%) had double mutations in *gyrA* (S83L and D87 to N or Y) with single mutation in *parC* (S80I), while 3 strains (15.8%) had double mutations in *gyrA* (S83L and D87 to N or Y) and *parC* (S80I and E84 to G or V). The positive efflux activity was linked to increased MIC values of ciprofloxacin (P < 0.001). Overall, the carriage of PMQR genes, efflux activity, and target mutations across *E. coli* strains contribute to ciprofloxacin resistance, a result that may necessitate a reassessment of the antibiotics in use for empirical UTIs therapy.

## Introduction

Most urinary tract infections (UTIs) are caused by bacteria in the order Enterobacterales, and *Escherichia coli* strains are responsable for 80% of uncomplicated UTIs, causing 95% and 50% of community and hospital-acquired UTIs, respectively. Additionally, *E. coli* is responsible for 80% of complicated UTIs [1]. Fluoroquinolones (FQs) like ciprofloxacin and levofloxacin should only be used to treat uncomplicated UTIs when there are no alternative treatment options available due to the risk of serious side effects. FQs may still have a role in treating complicated UTIs where the benefits outweigh the risks [2]. The misuse and overuse of FQs in outpatients are the main drivers in the development of FQ-resistant *E. coli*. The prevalence of FQ-resistant *E. coli* associated with UTIs in developing and developed countries is 56 − 85% and 5–32%, respectively [3], and the prevalence of FQ-resistant *E. coli* isolated from hospitalized patients with UTIs in Thailand was found to be as high as 65% [4].

FQs are broad-spectrum antimicrobial agents with higher efficacy against Gram-negative and Gram-positive bacteria than drugs in the quinolone group. They inhibit the activity of DNA gyrase and topoisomerase IV, blocking the progress of DNA synthesis. FQ resistance is generally caused by mutations in the quinolone resistance-determining regions (QRDRs) of the DNA gyrase (*gyrA* and *gyrB*) and/or topoisomerase IV genes (*parC* and *parE*) [5]. Other mechanisms are possible, including reducing drug diffusion into cells, driving drugs out of cells with efflux pumps, and acquiring plasmid-mediated quinolone resistance (PMQR) genes [6].

The incidence of PMQR is increasing and such resistance can be transferred to other bacterial cells through the process of horizontal gene transfer. The resistance genes found in PMQR include *qnr*, *aac(6′)-Ib-cr*, *qepA*, and *oqxAB* [7]. The *qnr* gene controls the production of pentapeptide repeat protein, which protects the enzyme DNA gyrase from inhibition by quinolones. It has different genetic variations and depends on genus, species, and strain of the bacteria. More than 100 families comprise the order Enterobacterales, divided into the 5 families *qnrA*, *qnrB*, *qnrC*, *qnrD*, and *qnrS* [8]. *Salmonella enterica* and *E. coli* are mostly found as types *qnrA*, *qnrB*, and *qnrS* [9]. The *aac(6′)-Ib-cr* gene encodes aminoglycoside acetyl transferase, modifying quinolones with a piperazinyl substituent like ciprofloxacin (CIP). The *oqxAB* and *qepA* genes encode the OqxAB and QepA efflux pumps to reduce susceptibility or resistance to FQ. Recently, *E. coli* strains containing the *oqxAB* gene have been identified as responsible for increasing the minimum inhibitory concentration (MIC) of CIP by an average of 7.5-fold (2–16-fold), while the *qepA* gene contributes to the increase in MIC of CIP by an average of 4.5-fold (2–31-fold) [10]. Although PMQR induces only low level resistance to FQ compared with the mechanism of mutation in the *gyrA* gene located on the bacterial chromosome [11], it has a crucial role in the transmission of resistance genes between bacterial cells and leads to the spread of antimicrobial resistant infections, posing an important public health threat.

Overexpression of resistance-nodulation-division (RND) efflux pumps results in resistance to quinolone, beta-lactam, chloramphenicol, tetracycline, and trimethoprim in bacteria [12]. *E. coli* is mostly found with the AcrAB-TolC efflux pump, which consists of three proteins, including an outer membrane protein TolC, an inner membrane transporter AcrB, and a periplasmic adapter protein AcrA. These proteins are encoded by *tolC, acrB*, and *acrA* genes, respectively [13]. High levels of FQ resistance in *E. coli* are correlated with increased expression of the AcrAB efflux pump by a gene located on the bacterial chromosome. The efflux pumps AcrAB and OqxAB serve an important function in multidrug resistance among *K. pneumoniae* strains [14]. Although the AcrAB, OqxAB, and QepA efflux pumps have been reported in many Gram-negative bacteria [10,15], the data on these efflux pump genes and their activity among FQ-resistant *E. coli* in Thailand are still questionable.

Therefore, the current study aimed to investigate the involvement of multiple mechanisms of FQ resistance in *E. coli* isolated from UTIs patients in Thailand.

## Materials and methods

### Bacterial strains and ethical approval

The current study included 131 ciprofloxacin-resistant *E. coli* isolates (MIC ≥ 2 μg/mL) from patients with UTIs at the Nopparat Rajathanee hospital between February and May 2018. Data on the phylogenetic groups of all isolates were previously published [4]. Using multiplex PCR, *E. coli* isolates were classified into groups A, B1, B2, C, D, E, F, and clade I based on the combined patterns of *arpA, chuA, yjaA*, TspE4.C2, *trpA, arpAgpE, trpAgpC*, and internal control genes [16]. Strains were classified into phylogenetic groups A (n = 4), B1 (n = 2), B2 (n = 84), C (n = 24), D (n = 1), E (n = 9), F (n = 5), and an unclassified group (n = 2). Ethical approval was granted by the Ethics Review Board (ERB) of the Research Institute, Rangsit University (DPE. No. RSUERB2022−019). The data access date is 7^th^ of February 2022. The data were anonymized, and no authors could identify individual patients during or after data collection.

### Detection of ESBLs production and multidrug resistance phenotype

Extended-Spectrum Beta-Lactamases (ESBLs) producing *E. coli* were confirmed by combination disk test. In brief, discs containing ceftazidime (30 μg), ceftazidime + clavulanic acid (30/10 μg), cefotaxime (30 μg), and cefotaxime + clavulanic acid (30/10 μg) (Oxoid, Hampshire, UK) were placed 30 mm apart (center to center) on a 0.5 McFarland lawn culture on a Mueller–Hinton agar plate (Oxoid, Hampshire, UK). The plates were incubated for 18–24 hours at 35°C. A positive result for ESBL production was indicated by an increase of ≥ 5 mm in the zone of inhibition when clavulanic acid was added [17]. *E. coli* ATCC 25922 and *Klebsiella quasipneumoniae* ATCC 700603 were used as the negative and positive control strains. Multidrug resistance (MDR) phenotype is generally defined as non-susceptibility to at least one agent in three or more different classes of antimicrobial agents [18].

### Assessment of MIC of fluoroquinolones

The MIC values of ciprofloxacin (Sigma-Aldrich, Burlington, MA, USA), levofloxacin and moxifloxacin (Glentham Life Sciences, Wiltshire, UK) in the *E. coli* strains were measured using a broth microdilution assay following the Clinical and Laboratory Standard Institute guideline [19]. *E. coli* ATCC 25922 was used as reference control strain. *E. coli* was prepared by colony suspension method to a quantity of approximately 10^8^ cells/mL and diluted to 1:100 in Mueller-Hinton broth (MHB) (Oxoid, Hampshire, UK), and 50 μL of the prepared culture was added into wells containing CIP, LEV, and MOX. The final concentrations of antibiotic in each well were between 2 and 1024 μg/mL. Plates were incubated at 37 °C for 16 − 18 hours before reading the MIC values.

### Measurement of efflux pump activity

The efflux pump activity of the *E. coli* strains was determined from the MICs of CIP with or without the efflux pump inhibitors (EPIs) Phe-Arg-β-naphthylamide (PAβN) or carbonyl cyanide 3-chlorophenylhydrazone (CCCP) (Sigma-Aldrich, Burlington, MA, USA). The efflux pump activity was examined using the broth microdilution assay following previously published methods [14,20]. Briefly, PAβN or CCCP were added to all the MHB wells containing 0.5–1.024 μg/mL ciprofloxacin at final concentrations of 20 μg/mL and 12.5 μg/mL, respectively. Wells containing PAβN or CCCP without ciprofloxacin were established as a control. For a strain to have positive efflux activity, the MIC value of ciprofloxacin without PAβN or CCCP must be at least 4-fold higher than that with PAβN or CCCP.

### DNA preparation for molecular analysis

The total genomic DNA was extracted from *E. coli* colonies grown on tryptone soya agar (Oxoid, Hampshire, UK) according to a previously described boiling method [21]. The purity and concentrations of DNA were assessed by agarose gel electrophoresis and Nano-400A (Allsheng, Hangzhou, China). The extracted DNA was stored at −20 °C until use.

### Screening of plasmid-mediated quinolone resistance

PMQR genes were detected among FQ-resistant *E. coli* strains by multiplex PCR, separated into 2 tubes. The first tube was analyzed for *qnrA*, *qnrB*, *qnrS*, and *acc(6’)-Ib-cr* genes and the second for *qnrC*, *qnrD*, *oqxAB*, and *qepA* genes. The PCR mixture (25 µL) contained 12.5 pmoles of each pair of primers (as shown in S1 Table), 12.5 µL of AccuStart II Geltrack PCR Supermix (Quantabio, Beverly, MA, USA) and approximately 100 ng of DNA template. Reaction mixture without DNA template was used as a negative control. PCR reactions were carried out using a Bio-Rad T100 Thermal Cycler (Hercules, CA, USA) under conditions described previously [22].

### Detection of AcrAB efflux pump genes

The *acrA*, *acrB*, and *tolC* genes were amplified using PCR with gene-specific primers (S1 Table). The primer sequences and PCR conditions were as previously described [13].

### Genetic analysis of QRDRs in *gyrA* and *parC* genes

Nineteen high level ciprofloxacin-resistant *E. coli* strains were chosen with different ciprofloxacin MICs for mutation analysis in the QRDRs of the *gyrA* and *parC* genes. Amplification was performed by PCR as previously described [23,24]. The primer sequences are listed in S1 Table.

### PCR analysis and DNA sequencing

The PCR product was analyzed using electrophoresis in 1.5% agarose gel and examined under a UV transilluminator. The gel with a size equivalent to the desired PCR product was cut and purified with a FavorPrep GEL/PCR Purification Kit (Favorgen, Ping Tung, Taiwan) before sending for DNA sequencing (Macrogen, Seoul, South Korea). Analysis and comparison of nucleotide sequences were performed using the BLAST program on the NCBI website (https://blast.ncbi.nlm.nih.gov/Blast.cgi).

### Nucleotide sequence accession number

The nucleotide sequences of *E. coli* strains analyzed in this study were submitted to GenBank under accession numbers PQ768832 to PQ768850 (*gyrA*), PQ768851 to PQ768869 (*parC*), PQ768870 to PQ768871 (*acrAB/tolC* efflux system), and PQ808976 to PQ808978 (PMQR genes).

### Statistical analysis

The data were statistically analyzed with IBM SPSS Statistics for Windows version 23.0 (IBM Corp., Armonk, NY, USA). Categorical variables were compared using the Chi-squared test or Fisher’s exact test. Comparisons of continuous variables were carried out using the Mann-Whitney U test or unpaired T-test. *P*-values ≤ 0.05 were considered statistically significant.

## Results

### Susceptibility to fluoroquinolones

Fluoroquinolone MIC distribution, MIC_50_ (MIC required to inhibit 50% of the strains), and MIC_90_ (MIC required to inhibit 90% of the strains) among 131 FQ-resistant *E. coli* strains are shown in Table 1. The MIC_50_ values of ciprofloxacin, levofloxacin, and moxifloxacin were 64, 16 and 16 µg/mL, respectively, and the MIC_90_ values were 256, 64 and 64 µg/mL, respectively. High-level ciprofloxacin resistance (MIC ≥ 32 µg/mL) was observed in 108 strains (82.4%). MIC_50_ and MIC_90_ of ciprofloxacin were higher than those of levofloxacin and moxifloxacin, indicating that these strains were most resistant to ciprofloxacin.

**Table 1:** Distribution of MIC (μg/mL) among fluoroquinolone-resistant *E. coli* strains.

Fluoroquinolones	Number of isolates (%) with indicated MIC value										MIC_50_	MIC_90_
MIC values (μg/mL)	2	4	8	16	32	64	128	256	512	1024	(µg/mL)	(µg/mL)
Ciprofloxacin	1 (0.8)	1 (0.8)	5 (3.8)	16 (12.2)	39 (29.8)	29 (22.1)	12 (9.2)	14 (10.7)	11 (8.4)	3 (2.3)	64	256
Levofloxacin	0 (0.0)	5 (3.8)	39 (29.8)	45 (34.4)	21 (16.0)	19 (14.5)	2 (1.5)	0 (0.0)	0 (0.0)	0 (0.0)	16	64
Moxifloxacin	1 (0.8)	1 (0.8)	17 (13.0)	51 (38.9)	29 (22.1)	21 (16.0)	8 (6.1)	3 (2.3)	0 (0.0)	0 (0.0)	16	64

### Prevalence of PMQR genes

Thirty-seven FQ-resistant *E. coli* strains (28.2%) were positive for PMQR genes by multiplex PCR assay. Among these strains, 33 (25.2%) and 4 (3.1%) harbored 1 and 2 PMQR genes, respectively (Table 2). The most frequent PMQR gene was *aac(6’)-Ib-cr* (30, 22.9%), followed by *qnrS* (10, 7.6%), and *qnrB* (1, 0.8%). The *qnrA*, *qnrC*, *qnrD*, *oqxAB*, and *qepA* genes were not observed in the current study. Of the total 131 FQ-resistant *E. coli* strains, 72 (55.0%) were ESBL producers, while 34 (26.0%) exhibited the MDR phenotype. The presence of PMQR genes was significantly related with the ESBLs and MDR phenotype of the strains (Chi-square, *P*-value < 0.001).

**Table 2:** Profiles of PMQR gene and FQ MIC levels in fluoroquinolone-resistant *E. coli* strains.

No. of genes	PMQR profiles	No. of isolates (%)	MIC_50_ (µg/mL)		
			CIP	LEV	MOX
1 gene, n = 33 (25.2%)					
	*aac(6’)-Ib-cr*	26 (19.9)	256	16	32
	*qnrS*	7 (5.3)	128	64	128
2 genes, n = 4 (3.1%)					
	*aac(6’)-Ib-cr, qnrS*	3 (2.3)	256	64	128
	*aac(6’)-Ib-cr, qnrB*	1 (0.8)	64	32	64

The PMQR profiles and MIC values of FQ in *E. coli* strains were also investigated. The presence of *aac(6’)-Ib-cr* and *qnrS* genes was detected in three strains (2.3%), and *aac(6’)-Ib-cr* with *qnrB* was observed in one strain (0.8%). Increased levofloxacin and moxifloxacin MICs were observed in strains exhibiting coexistence of genes *aac(6’)-Ib-cr* and *qnrS* or *qnrB* when compared with isolates carrying the *aac(6’)-Ib-cr* gene alone (Table 2).

### Efflux pump activity

**Fig 1:**
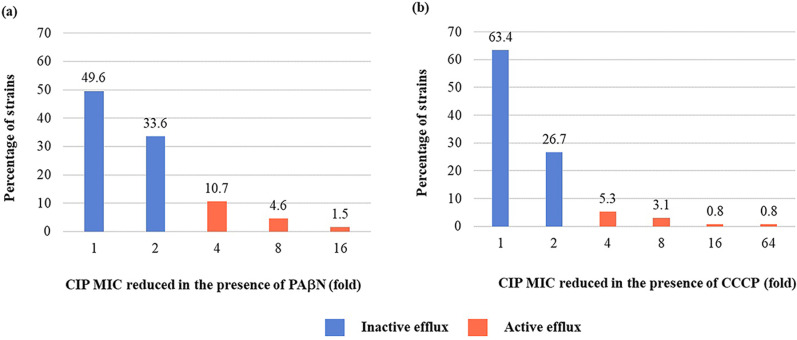
Percentage of strains showing efflux pump activity among fluoroquinolone-resistant *E. coli.* Ciprofloxacin MIC reduced in the presence of PAβN **(a)** and CCCP **(b)** as efflux pump inhibitors. Strains with ciprofloxacin MIC reduced ≥ 4-fold are considered to have positive efflux activity. Here, MIC = Minimum inhibitory concentration, CIP = ciprofloxacin, PAβN = Phe-Arg-β-naphthylamide, CCCP = carbonyl cyanide 3-chlorophenylhydrazone.

Efflux pump inhibition reduced ciprofloxacin MIC by at least 4-fold in 33 (25.2%) of FQ-resistant *E. coli* strains. Comparison of ciprofloxacin MIC values in the presence and absence of PAβN revealed a significant reduction from 4- to 16-fold after using PAβN in 22 isolates (16.8**%)**. Among these former strains, 2 (1.5**%)** showed 16-fold reduction in MIC, 6 (4.6**%)** showed 8-fold reduction, and 14 strains (10.7**%)** showed 4-fold reduction in MIC. On the other hand, 44 strains (33.6%) exhibited 2-fold reduction in ciprofloxacin MIC whereas ciprofloxacin MIC of 65 strains (49.6%) did not show any difference when combined with PAβN (Fig. 1a). When using CCCP, a decrease in ciprofloxacin MIC value was observed from 4- to 64-fold in 13 strains (9.9%). Among these former strains, 1 (0.8%) showed 64-fold and 16-fold reduction in MIC, 4 (3.1%) showed 8-fold, and 7 (5.3%) revealed 4-fold reduction in MIC. In the negative efflux activity group, 35 strains (26.7%) showed a 2-fold reduction in MIC and 83 (63.4%) showed no difference in MIC in the presence or absence of CCCP (Fig. 1b).

Higher ciprofloxacin, levofloxacin, and moxifloxacin MIC_50_ were found among active efflux strains (n = 33) compared to inactive strains (n = 98) (MIC_50_: 256 vs. 32 µg/mL, 32 vs. 16 µg/mL, and 32 vs. 16 µg/mL, respectively). The ciprofloxacin, levofloxacin, and moxifloxacin MIC values in the active efflux group were statistically significantly higher than in the inactive group (Mann Whitney U test; ciprofloxacin P-value < 0.001, levofloxacin and moxifloxacin P-value = 0.015) (Table 3).

**Table 3:** Fluoroquinolone MIC values between active and inactive efflux fluoroquinolone-resistant *E. coli* strains.

Fluoroquinolones	Active efflux strains (n = 33)			Inactive efflux strains (n = 98)			P-value
	MIC range	MIC_50_	MIC_90_	MIC range	MIC_50_	MIC_90_	
Ciprofloxacin	8 − 1024	256	512	2 − 512	32	128	< 0.001
Levofloxacin	4 − 128	32	64	4 − 128	16	64	0.015
Moxifloxacin	8 − 128	32	128	2 − 256	16	64	0.015

### MIC values are presented in µg/mL

#### Molecular detection of chromosomal efflux pump genes.

PCR assay using specific primers for *acrA*, *acrB*, and *tolC* genes demonstrated that all FQ-resistant *E. coli* strains (131, 100%) carried those genes and consequently comprise the AcrAB efflux system. The expected PCR product size of *acrA*, *acrB*, and *tolC* genes were 1194, 761, and 1170 base pairs, respectively. Sequence analysis revealed that these PCR products corresponded to those genes of *E. coli*.

#### GyrA and ParC mutations.

The amino acid mutation profiles in QRDRs of GyrA and ParC in 19 high-level ciprofloxacin-resistant *E. coli* strains, along with phylogenetic group, ciprofloxacin MIC values, efflux activity, and PMQR determinants, are detailed in Table 4. All strains had double missense mutations in codon 83 (Ser to Leu) and 87 (Asp to Asn/Tyr) in GyrA and single substitution at codon 80 (Ser to Ile) in ParC. Another amino acid substitution in ParC was evident at codon 84 (Glu to Val/Gly). Triple mutations (2 GyrA/1 ParC) were predominantly found in the tested strains (16/19, 84.2%) followed by quadruple mutations (2 GyrA/2 ParC) in 15.8% (3/19). There was no significant difference in ciprofloxacin MIC among the groups of strains with different numbers of target site mutations (Mann Whitney U test; P-value = 0.210). The highest ciprofloxacin MIC_50_ (128 µg/mL) was shown by the group of strains containing triple mutations, whereas those carrying quadruple mutations had ciprofloxacin MIC_50_ at 512 µg/mL. Three strains harboring quadruple mutations were classified into phylogenetic group B2, and two of them (EC33 and EC29) had positive efflux activity contributing to an increase in ciprofloxacin MIC (≥ 512 µg/mL). In contrast, EC57, carrying quadruple mutations and negative efflux activity, revealed a lower ciprofloxacin MIC when compared to EC33 and EC29.

**Table 4:** Phylogenetic group, ciprofloxacin MICs, efflux activity, PMQR genes, and QRDR mutations among 19 high level ciprofloxacin resistant E. coli strains.

Strain No.	Phylogenetic group	CIP MIC (µg/mL)	Efflux activity	PMQR genes	QRDRs mutations^a^	
					GyrA	ParC
EC47	B2	32	+	–	S83L, D87N.	S80I.
EC76	E	32	−	–	S83L, D87N.	S80I.
EC57	B2	64	−	*qnrS*	S83L, D87Y.	S80I, E84G.
EC136	B2	64	−	*aac(6’)-Ib-cr*	S83L, D87N.	S80I.
EC142	C	64	−	*aac(6’)-Ib-cr, qnrB*	S83L, D87N.	S80I.
EC92	C	64	−	–	S83L, D87N.	S80I.
EC182	F	64	+	–	S83L, D87Y.	S80I.
EC225	B1	128	−	*qnrS*	S83L, D87N.	S80I.
EC87	B2	128	+	–	S83L, D87N.	S80I.
EC226	C	128	+	*qnrS*	S83L, D87N.	S80I.
EC186	E	128	−	–	S83L, D87N.	S80I.
EC116	C	256	−	*aac(6’)-Ib-cr*, *qnrS*	S83L, D87N.	S80I.
EC117	E	256	+	–	S83L, D87N.	S80I.
EC156	E	256	+	–	S83L, D87N.	S80I.
EC99	F	256	+	*aac(6’)-Ib-cr*	S83L, D87N.	S80I.
EC110	C	512	+	–	S83L, D87N.	S80I.
EC209	C	512	−	*aac(6’)-Ib-cr*	S83L, D87N.	S80I.
EC33	B2	512	+	*aac(6’)-Ib-cr*	S83L, D87N.	S80I, E84V.
EC29	B2	1024	+	–	S83L, D87N.	S80I, E84V.

CIP MIC, ciprofloxacin MIC (µg/mL); PMQR, plasmid-mediated quinolones resistance; QRDRs, quinolone resistance-determining regions; ^a^ QRDRs of *E. coli* str. K-12 substr. MG1655 (accession number: NC_000913) as a wild-type reference.

### Association between distribution of phylogenetic group, PMQR genes, and efflux activity

A significant frequency was observed of the *qnrS* gene, and PMQR determinants in *E. coli* strains across phylogenetic groups C (6, 4.6%) and B2 (18, 13.7%) (Fisher’s exact test, P = 0.022 vs. P = 0.039) (Table 5). In contrast, the percentage of *aac(6’)-Ib-cr* and *qnrB* genes among phylogenetic groups was not significantly different (P = 0.252 and P = 0.359, respectively). Genes *aac(6’)-Ib-cr* and *qnrB* were most frequently detected in phylogenetic group B2 (16, 12.2%), and *qnrB* in group C (1, 0.8%). The co-carriage of *aac(6’)-Ib-cr* with *qnrS* or *qnrB* genes were predominantly found in group C (3, 2.3%).

**Table 5:** Association between frequency of PMQR genes, efflux pump activity, and phylogenetic groups in fluoroquinolone-resistant E. coli strains.

PMQR genes	A (n = 4)	B1 (n = 2)	B2 (n = 84)	C (n = 24)	D (n = 1)	E (n = 9)	F (n = 5)	Unassignable (n = 2)	*P*-value
*aac(6’)-Ib-cr*, n (%)	0 (0.0)	0 (0.0)	16 (12.2)	8 (6.1)	0 (0.0)	3 (2.3)	3 (2.3)	0 (0.0)	0.252
*qnrS,* n (%)	0 (0.0)	1 (0.8)	3 (2.3)	6 (4.6)	0 (0.0)	0 (0.0)	0 (0.0)	0 (0.0)	**0.022**
*qnrB,* n (%)	0 (0.0)	0 (0.0)	0 (0.0)	1 (0.8)	0 (0.0)	0 (0.0)	0 (0.0)	0 (0.0)	0.359
PMQR determinants, n (%)	0 (0.0)	1 (0.8)	18 (13.7)	12 (9.2)	0 (0.0)	3 (2.3)	3 (2.3)	0 (0.0)	**0.039**
**Efflux activity**									
Active with PAβN, n (%)	0 (0.0)	0 (0.0)	5 (3.8)	5 (3.8)	1 (0.8)	6 (4.6)	4 (3.1)	1 (0.8)	**< 0.001**
Active with CCCP, n (%)	0 (0.0)	1 (0.8)	7 (5.3)	3 (2.3)	0 (0.0)	1 (0.8)	0 (0.0)	1 (0.8)	0.242
Active with PAβN or CCCP, n (%)	0 (0.0)	1 (0.8)	12 (9.2)	8 (6.1)	1 (0.8)	6 (4.6)	4 (3.1)	1 (0.8)	**< 0.001**

The relative frequency of positive efflux activity with PAβN or CCCP among *E. coli* phylogenetic groups was significantly different (Fisher’s exact test, P **< 0.00**1) (Table 5). This efflux activity was more statistically abundant in phylogenetic groups B2 (12, 9.2%**).**

## Discussion

Resistance rates in *E. coli*, the most frequent pathogen in UTIs, to FQs such as ciprofloxacin are reported to be greater than 20% [25]. In this study, FQ resistance mechanisms in *E. coli* strains from patients with UTI were investigated, with 131 FQ-resistant *E. coli* strains included. Most strains (82.4%) exhibited high ciprofloxacin resistance using the broth microdilution method, in agreement with previous reports on the prevalence of high-level ciprofloxacin resistance in *Enterobacteriaceae* isolates in South Africa and Egypt [24,26], and *E. coli* strains in Iran, Bangladesh, and Kenya [27–29]. Increasing ciprofloxacin MIC values may cause unreasonable ciprofloxacin use, and horizontal and vertical transmission of the genotype within and among hosts. Our findings show that levofloxacin and moxifloxacin have lower MIC_50_ and MIC_90_ than ciprofloxacin due to their structures that contain a C8-methoxy group that improves their action against *E. coli* with topoisomerase mutations relative to wild-type, parental strains [30, 31].

The main mechanism of FQ resistance involves point mutations in the target enzyme-coding genes. PCR and DNA sequencing based on the QRDR sequences of the *gyrA* and *parC* genes of the chosen strains from diverse FQ-resistant *E. coli* demonstrated that all strains possessed missense mutations in *gyrA* at codon 83 (Ser to Leu) and 87 (Asp to Asn/Tyr), which are located near the active site of DNA gyrase. Mutations at these active sites may affect the binding of FQ to the site, contributing to reduced susceptibility or resistance to FQ [32]. These are the most commonly found mutations worldwide [5]. In the *parC* gene, all strains showed mutations at codon 80 (Ser to Ile) and some strains also at codon 84 (Glu to Val/Gly), which are those most commonly found in previous reports [24,32 − 34]. All strains showed at least three target site mutations (2 *gyrA*/1 *parC*) or (2 *gyrA*/2 *parC*), conferring high level ciprofloxacin resistance among *E. coli* strains. In previous studies, these mutation profiles were those most often identified among ciprofloxacin-resistant *E. coli.* [24,33 − 34]. In addition, this finding confirms studies indicating that double mutations in *gyrA* in *E. coli* were necessary to confer high-level fluoroquinolone resistance, with mutation in *parC* contributing as the second step [23,35]. Our findings demonstrate that three and four target site mutations had no significant effect on ciprofloxacin MIC among the tested strains (P = 0.210). This difference could indicate additional mechanisms of fluoroquinolone resistance, including harbored PMQR genes, upregulation of efflux pumps, and reduced membrane permeability [6].

PMQR genes play a crucial role in decreased susceptibility to FQ and allow some low-fitness mutants below the resistance breakpoint to develop a higher level of resistance against FQ by just one or two amino acid mutations in the QRDRs [36]. The prevalence of PMQR genes in this study was 28.2%, higher than that reported in Taiwan (14.9%) [37], and South Korea (10.7%) [38], and lower than in Iran (90%) [27], Kenya (70.7%) [29], and Nepal (51.5%) [39]. We found prevalence of the three PMQR genes *aac(6’)-Ib-cr* (n = 30, 22.9%), *qnrS* (n = 10, 7.6%), and *qnrB* (n = 1, 0.8%). The most abundant PMQR gene among FQ-resistant *E. coli* was *aac(6’)-Ib-cr*, consistent with previous studies in China (19.7%) [40], Iran (72.0%) [27], South Korea (9.0%) [38], Nepal (31.2%) [39], and Kenya (47.0%) [29]. For plasmid-mediated efflux pumps, *qepA* and *oqxAB* genes were not detected. Similar findings were reported in Tunisia [41] and Egypt [24].

PMQR genes, including *qnr* genes, function by protecting the target enzymes from ciprofloxacin inhibition. Efflux pump genes, such as *qepA* and *oqxAB*, work by actively pumping the drug out of the bacterial cell. In contrast, the *aac(6’)-Ib-cr* gene encodes a variant of aminoglycoside acetyltransferase, which possesses a unique capability to modify ciprofloxacin. The AAC(6’)-Ib-cr enzyme mediates the N-acetylation of the piperazinyl amine group on the ciprofloxacin molecule. It adds an acetyl group to the nitrogen atom within the piperazine ring of ciprofloxacin. This modification reduces the drug’s ability to effectively bind to its target enzymes, DNA gyrase and topoisomerase IV, decreasing its antimicrobial activity [42–43]. Despite levofloxacin containing a piperazinyl group, studies have revealed that the AAC(6′)-Ib-cr enzyme has a greater effect on the MICs of ciprofloxacin and norfloxacin than on the MIC of levofloxacin [30,44]. While moxifloxacin lacks this piperazinyl group, it is generally considered to be less susceptible to modification by the AAC(6′)-Ib-cr enzyme [42]. These reasons strongly support our finding that *E. coli* isolates carrying the *aac(6’)-Ib-cr* gene showed a greater increase in the MIC_**50**_ for ciprofloxacin compared to the MIC_**50**_ values for levofloxacin and moxifloxacin (Table 2). Moreover, AAC(6’)-Ib-cr is a bifunctional enzyme, which can also modify aminoglycoside antibiotics [43]. This dual activity contributes to the spread of resistance to multiple classes of antibiotics.

In general, *aac(6’)-Ib-cr* and *qnrB* or *qnrS* are the most prevalent co-existing PMQR genes among Enterobacterales. We found that 3.1% of the strains carried *aac(6’)-Ib-cr* and *qnrB* or *qnrS*, consistent with recent reports in Nepal [39] and Kenya [29]. The frequency and distribution of PMQR genes vary among studies, which could be attributed to the study population, isolate selection criteria, and type of PMQR genes detected. The AAC(6’)-Ib-cr enzyme and Qnr protein confers significantly decreased susceptibility to ciprofloxacin, with MICs increasing 3- to 4-fold and 8- to 32-fold, respectively, when compared to the wild-type susceptibility profile [42,45]. In contrast, MICs of FQs among strains co-carrying *aac(6’)-Ib-cr* and *qnrB* or *qnrS* and single PMQR gene-positive strains were likely not to be different (Mann Whitney U test; P-value = 0.900).

In the current study, the prevalence of ESBLs production and MDR phenotype among FQ-resistant *E. coli* strains are relatively lower than in studies in India [46], Iran [47], and Egypt [33]. However, ESBLs production and MDR phenotype are linked to the presence of PMQR genes (P < 0.001), which may be because the plasmids harbor genes encoding both ESBLs and multi-resistance to various antibiotics, including fluoroquinolones. This highlights the clinical importance of these plasmid-transmitted genes.

To elucidate the effect of the efflux pump in ciprofloxacin resistance in *E. coli* strains, PAβN and CCCP were used to measure the activity of the pump. Based on the 4-fold or greater decrease in ciprofloxacin MIC after augmentation of PAβN or CCCP, efflux activity was found in 16.8 and 9.9% of the strains, respectively. This discrepancy may be due to different EPI mechanisms. Binding of PAβN to functional efflux pumps reduces the ability of the pumps to interact with antibiotics. CCCP disrupts the proton gradient of the membrane, inhibiting the activation of the RND family of efflux pumps, including the AcrAB pump [48]. The AcrAB and OqxAB efflux pumps in the Enterobacterales order, particularly *E. coli*, have been investigated for their involvement in ciprofloxacin resistance [10,48,^49^]. PCR assay showed that all strains had *acrA*, *acrB*, and *tolC* chromosomal efflux genes but the plasmid-mediated *qepA* and *oqxAB* efflux genes were not detected. Overall, active efflux in the current study was 25.2%, confirming the role of the AcrAB efflux system in ciprofloxacin resistance among these strains. On the other hand, the remaining 74.8% of strains were efflux inactive but carried *acrA*, *acrB*, and *tolC*. A possible explanation for this is that these strains do not express efflux pump genes. To validate this, further evaluation of the expression of genes that encode efflux pumps is required, including the transcriptional regulator genes such as *marR*, *acrR*, and *soxS* [50,^51^].

Our findings show that FQ MIC values are significantly increased among strains showing positive efflux activity, compared to negative strains. The MIC_50_ of ciprofloxacin, levofloxacin, and moxifloxacin among active efflux strains were 256, 32, and 32 µg/mL, respectively, indicating that the efflux pump is related to high-level FQ resistance. Two strains, EC33 and EC29, that have four target site mutations (2 *gyrA*/2 *parC*), with positive efflux activity induced ciprofloxacin MIC of 512 and 1024 µg/mL. However, among highly ciprofloxacin-resistant strains, the ciprofloxacin MIC values did not differ significantly between strains with target mutations in gyrA and parC combined with active efflux, and those with target mutations combined with PMQR gene carriage (Mann Whitney U test; P-value = 0.511). Positive efflux activity was not associated with the MDR phenotype (P = 0.262). It is possible that the observed MDR phenotype carried multiple plasmids rather than the active efflux pump, which is more commonly found in the order Enterobacterales, unlike the glucose non-fermenter in which the main mechanism of MDR was found to be the efflux system [5,24].

The phylogenetic groups of our strains were previously characterized, identifying group C with the highest rates of ciprofloxacin, norfloxacin, and levofloxacin resistance [4]. In the current study, the distribution of PMQR genes was significantly different across phylogenetic groups. Notably, group C was found to have greater PMQR genes diversity and a higher relative frequency of *qnrS*. In addition, the co-carriage of *aac(6’)-Ib-cr* and *qnrB* or *qnrS* positive strains belonged to group C (2.3%). Group C has been isolated from various environmental samples including the gut microbiota of animals and humans [52–53]. The presence of group C in various habitats is probably linked to the capacity of its different lineages to obtain new genes via horizontal transfer, enabling them to adapt to specific environments, such as those with antibiotic presence. This adaptability may account for the observed diversity in PMQR genes within the group. PMQR determinants showed a significant association with group B2, consistent with the study in Iran which revealed a higher occurrence of PMQR genes in group B2 [54]. Group B2 is known to be predominantly associated with extraintestinal infections in human including UTIs and often exhibits higher virulence [55]. The higher PMQR gene frequency in group B2 is likely a result of a combination of factors, including increased antibiotic selection pressure due to their role in human infections, their tendency to acquire and maintain mobile genetic elements carrying resistance genes, the clonal spread of resistant and virulent strains, and potential linkages between virulence and resistance determinants. To better understand how antimicrobial resistance develops and spreads within a population, a more accurate method like sequence type analysis is available; therefore, additional comprehensive research on this issue will be required. Efflux pump activity conferring reduced ciprofloxacin MIC was found predominantly in group B2. The PMQR genes and efflux activity were not detected in group A. Therefore, mutation in QRDRs and decreased expression of outer membrane porins may be the mechanism of ciprofloxacin resistance in this group.

A strength of the current study is that we demonstrated multiple mechanisms of FQ resistance in *E. coli* isolates from patients with urinary tract infections in Thailand. We successfully used PAβN and CCCP, two different efflux pump inhibitors, to quantify ciprofloxacin efflux activity in these isolates. Specifically, we report the efflux activity against ciprofloxacin associated with AcrAB efflux pump. However, this study had some limitations. Despite the significant number of isolates analyzed in this study, its limited scope means it might not fully reflect the geographic and population variations found throughout Thailand. To overcome this, future research should include a wider and more geographically diverse selection of isolates from various population groups in Thailand to improve how broadly the findings can be applied. Mutations in gyrase A and topoisomerase genes in all FQ-resistant *E. coli* strains were not analyzed due to limited resources. In addition, permeability alteration as a mechanism conferring FQ resistance was not investigated.

## Conclusions

Our study has highlighted the alarmingly high-level of FQ resistance among *E. coli* strains in Thailand. We have confirmed that the harboring of PMQR genes, efflux activity, and *gyrA* and *parC* mutations are involved in conferring FQ resistance. The *aac(6’)-Ib-cr* gene was the most prevalent among the PMQR genes and the *qnr* gene remained rare. A correlation was observed between the occurrence of active efflux and increased FQ MIC levels. Our findings provide a better understanding of the molecular mechanisms of FQ resistance in *E. coli* strains and should contribute to more effective strategies for treating UTIs patients and preventing the spread of antimicrobial resistance bacteria.

## Supporting information

S1 Table Oligonucleotide primers used for PCR amplification. (DOCX)
