# Supplementary material for: Mechanisms of fluoroquinolone resistance among Escherichia coli isolates from urinary tract infections in Thailand
Source: PLoS One. 2025 May 30;20(5):e0325175. doi: 10.1371/journal.pone.0325175 (PMC12124560; doi:10.1371/journal.pone.0325175)
Supplement: S1 Table — (DOCX) [file pone.0325175.s001.docx]

**S1 Table.** Oligonucleotide primers used for PCR amplification.

| **Target gene** | **Primer** | **5’-3’ primer sequence** | **T_a_** **(°C)** | **Amplicon size (bp)** | **Reference** |
| --- | --- | --- | --- | --- | --- |
| *gyrA* | F  R | CGACCTTGCGAGAGAAAT GTTCCATCAGCCCTTCAA | 55 | 620 | 1 |
| *parC* | F  R | CGTGCGTTGCCGTTTATTG ATCTTCTTTCTTCCACACCGC | 60 | 663 | 2 |
| *qnrA* | F  R | CAGCAAGAGGATTTCTCACG  AATCCGGCAGCACTATTACTC | 63 | 630 | 3 |
| *qnrB* | F  R | GGCTGTCAGTTCTATGATCG  GAGCAACGATGCCTGGTAG | 63 | 488 |  |
| *qnrS* | F  R | GCAAGTTCATTGAACAGGGT  TCTAAACCGTCGAGTTCGGCG | 63 | 428 |  |
| *aac(6’)-Ib-cr* | F  R | TTGGAAGCGGGGACGGAM  ACACGGCTGGACCATA | 63 | 260 |  |
| *qnrC* | F  R | GCAGAATTCAGGGGTGTGAT  AACTGCTCCAAAAGCTGCTC | 63 | 118 |  |
| *qnrD* | F  R | CGAGATCAATTTACGGGGAATA AACAAGCTGAAGCGCCTG | 63 | 581 |  |
| *oqxAB* | F  R | CCGCACCGATAAATTAGTCC  GGCGAGGTTTTGATAGTGGA | 63 | 313 |  |
| *qepA* | F  R | GCAGGTCCAGCAGCGGGTAG  CTTCCTGCCCGAGTATCGTG | 63 | 218 |  |
| *acrA* | F  R | CAATTTGAAATCGGACACTCG  GGCATGTCTTAACGGCTCCT | 55 | 1194 | 4 |
| *acrB* | F  R | GAAAGGCCAACAGCTTAAC GAGCTGGAGTCAGGATCAAC | 65 | 761 |  |
| *tolC* | F  R | TGCTCCCCATTCTTATCGGC  GCTCTTGCTTGGCGTTGTAC | 65 | 1170 |  |

**References**

1. Weigel LM, Steward CD, Tenover FC. gyrA mutations associated with fluoroquinolone resistance in eight species of *Enterobacteriaceae.* Antimicrob Agents Chemother. 1998; 42(10):2661-7. doi: 10.1128/AAC.42.10.2661. PMID: 9756773.

2. Hamed SM, Elkhatib WF, El-Mahallawy HA, Helmy MM, Ashour MS, Aboshanab KMA. Multiple mechanisms contributing to ciprofloxacin resistance among Gram negative bacteria causing infections to cancer patients. Sci Rep. 2018; 8(1):12268. doi: 10.1038/s41598-018-30756-4. PMID: 30115947.

3. Ciesielczuk H, Hornsey M, Choi V, Woodford N, Wareham DW. Development and evaluation of a multiplex PCR for eight plasmid-mediated quinolone-resistance determinants. J Med Microbiol. 2013; 62(Pt 12):1823-1827. doi: 10.1099/jmm.0.064428-0. Epub 2013 Sep 2. PMID: 24000223.

4. Chowdhury N, Suhani S, Purkaystha A, Begum MK, Raihan T, Alam MJ, Islam K, Azad AK. Identification of AcrAB-TolC Efflux Pump Genes and Detection of Mutation in Efflux Repressor AcrR from Omeprazole Responsive Multidrug-Resistant *Escherichia coli* Isolates Causing Urinary Tract Infections. Microbiol Insights. 2019 Dec; 12:1178636119889629. doi: 10.1177/1178636119889629. PMID: 31839709.
